# Hypertension and concomitant arteriosclerotic diseases are risk factors for colonic diverticular bleeding: a case–control study

**DOI:** 10.1007/s00384-012-1422-x

**Published:** 2012-02-22

**Authors:** Ryota Niikura, Naoyoshi Nagata, Junichi Akiyama, Takuro Shimbo, Naomi Uemura

**Affiliations:** 1 Department of Gastroenterology and Hepatology, National Center for Global Health and Medicine, 1-21-1 Toyama, Shinjuku, 162-8655 Tokyo, Japan; 2 Department of Clinical Research and Informatics, National Center for Global Health and Medicine, 1-21-1 Toyama, Shinjuku, 162-8655 Tokyo, Japan; 3 Department of Gastroenterology and Hepatology, Kohnodai Hospital, National Center for Global Health and Medicine, 1-7-1 Kohnodai, Ichikawa, 272-8516 Chiba, Japan

**Keywords:** Arteriosclerotic diseases, Colonic diverticular bleeding, Colonic diverticulosis, Risk factors

## Abstract

**Purpose:**

Colonic diverticular bleeding is a major cause of lower gastrointestinal bleeding. However, a limited number of studies have been reported on the risk factors for diverticular bleeding. Our aim was to identify risk factors for diverticular bleeding.

**Methods:**

Our study design is a case (diverticular bleeding)–control (diverticulosis) study. We prospectively collected information of habits, comorbidities, history of medications and symptoms by a questionnaire, and diagnosed diverticular bleeding and diverticulosis by colonoscopy. Logistic regression models were used to estimate odds ratio (OR) and 95% confidence interval (CI).

**Results:**

A total of 254 patients (diverticular bleeding, 45; diverculosis, 209) were selected for analysis. Cluster (≥10 diverticula) type (OR, 4.0; 95% CI, 1.8–8.9), hypertension (OR, 2.2; 95% CI, 1.0–4.6), ischemic heart disease (OR, 2.4; 95% CI, 1.1–5.4), and chronic renal failure (OR, 6.4; 95% CI, 1.3–32) were independent risk factors for diverticular bleeding.

**Conclusions:**

Large number of diverticula, hypertension, and concomitant arteriosclerotic diseases including ischemic heart disease and chronic renal failure are risk factors for diverticular bleeding. This study identifies new information on the risk factors for diverticular bleeding.

## Introduction

Colonic diverticula are pseudodiverticula resulting from herniation of the mucosa and submucosa through a weakened portion of the colonic wall [1]. Colonic diverticulosis is an acquired disease caused by increased intestinal pressure due to decreased intake of dietary fiber and mucosal fragility associated with aging [2–5]. The typical locations of colonic diverticula differ between the Western and Asian populations, appearing predominantly left-sided in the Western countries and predominately right-sided in Asia [6]. With changes in dietary habits and the aging of society, the incidence of colonic diverticulosis is expected to increase [3, 4].

Although colonic diverticulosis is usually asymptomatic, diverticulitis and diverticular bleeding can occasionally occur. In particular, colonic diverticular bleeding causes sudden, painless hematochezia, and with massive bleeding, becomes a condition with high morbidity and mortality rates, in which blood transfusion and urgent treatment are required [7]. Bleeding develops in 3–5% of patients with colonic diverticulosis, and surgical intervention is required in 10–30% of the cases [8, 9]. In addition, patients with colonic diverticular bleeding show a high rate of recurrence within a short period [10]. With the increase in colonic diverticulosis, an increase in diverticular bleeding is to be expected [11]. However, the pathogenesis of bleeding remains unclear [12, 13]. Hypertension and use of nonsteroidal anti-inflammatory drugs (NSAIDs) have been reported as risk factors [12–14], but these issues have yet to be extensively investigated.

Accurate accumulation of data is important to evaluate the risk factors. Previous reports have investigated medical records retrospectively and thus may have been influenced by recall bias. We prospectively evaluated data including comorbidities, medications, and locations of diverticula. The purpose of this study was to identify risk factors for colonic diverticular bleeding.

## Methods

### Study design

We prospectively collected information of habits, comorbidities, history of medications, type of diverticula, and symptoms. We performed a case (diverticular bleeding)–control (diverticulosis) study to identify the risk factors for colonic diverticular bleeding.

### Patient selection

**Fig. 1:**
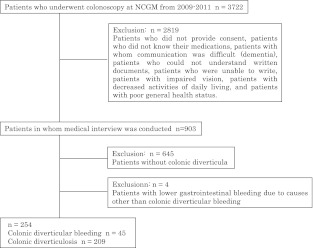
Patient selection

Eligibility criteria included patients who underwent colonoscopy at the National Center for Global Health and Medicine (NCGM) between November 2009 and February 2011. NCGM is a tertiary care academic center with 900 beds, located in metropolitan Tokyo. Exclusion criteria were as follows: patients who did not provide informed consent, patients who did not know what medications they were receiving, patients with whom communication was difficult, patients who could not understand written documents, patients who were unable to write, patients with impaired vision, patients with decreased activities of daily living, patients who were unable to respond to the questionnaire due to serious illness, patients without diverticula in the colon on colonoscopy, patients in whom total colonoscopy could not be performed, and patients with lower gastrointestinal (GI) bleeding due to causes other than colonic diverticular bleeding (Fig. 1).

### Diagnosis

**Fig. 2:**
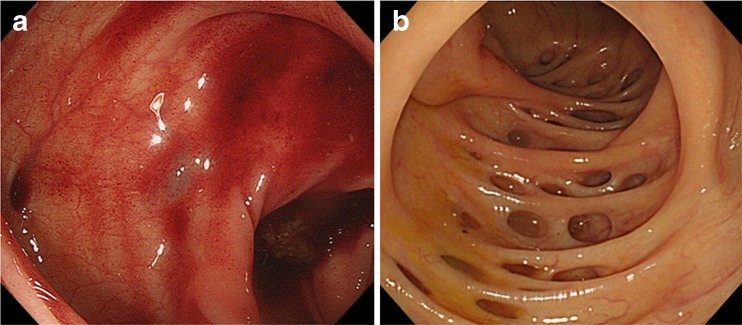
**a** Colonic diverticular bleeding. **b** Colonic diverticulum

An electronic video endoscope (high-resolution scope, model CFH260; Olympus Optical, Tokyo, Japan) was used for the diagnosis of colonic diverticulosis and colonic diverticular bleeding (Fig. 2). Intestinal lavage for endoscopic examination was performed using 2 L of solution containing polyethylene glycol. Diagnosis of colonic diverticular bleeding was based on the criteria reported by Jensen [15], including a chief complaint of painless hematochezia and the exclusion of hemorrhoidal bleeding on anal examination. We assessed the location and type of diverticula using colonoscopy. Location was defined as right-sided, involving the transverse or proximal colon; left-sided, involving the descending or distal colon; or bilateral, around the entire colon. Type was defined as sporadic type for 1 diverticulum, scatter type for 2–9 diverticula, and cluster type for ≥10 diverticula.

### Questionnaire

The questionnaire included lifestyle habits, comorbid diseases, medications, and symptoms. Medical history was obtained in a face-to-face interview with medical staff. For the medication history, prescriptions and medical records were reviewed in addition to information from the patients themselves, in order to avoid omissions.

#### Habits

Smoking and alcohol drinking habits were inquired about. A smoker was defined as someone who smoked at the time of, or anytime prior to, the interview. A drinker was defined as someone who consumed alcohol at least 1 day/week.

#### Comorbidities

Comorbidities that were evaluated included hypertension, diabetes mellitus, hyperlipidemia, ischemic heart disease, chronic liver dysfunction, and chronic renal failure. Hypertension was considered present in patients taking any antihypertensive drugs. Diabetes mellitus was defined based on the diagnostic criteria of the American Diabetes Association [16]. Hyperlipidemia was considered present in patients taking any antihyperlipidemic drugs. Ischemic heart disease was considered present in any patient with a history of myocardial infarction or angina pectoris. Chronic liver dysfunction included chronic viral hepatitis and alcoholic liver disease. Chronic renal failure was considered present in patients on hemodialysis or peritoneal dialysis, or with serum creatinine ≥2.0 mg/dL.

#### History of medications

Patients were asked about the use of antiplatelet drugs (aspirin, clopidogrel, cilostazol, ticlopidine), anticoagulants, acetaminophen, NSAIDs, steroids, and antihypertensive drugs. The survey form included photographs of all these oral drugs, which are approved in Japan. Use of a medication was defined as oral administration starting at least 1 month before the interview.

#### Symptoms of constipation

Symptoms of constipation were evaluated in 7 grades using the subscale of Gastrointestinal Symptom Rating Scale [25]: 1, no impediment to daily activities; and 7, symptoms so severe as to be intolerable. Positive symptoms were defined as a score ≥3.

### Ethics

The study protocol was approved by the NCGM Ethics Committee. Written informed consent was obtained from all patients prior to starting the study. The study protocol was registered with UMIN 000004533.

### Statistics

Patients with colonic diverticular bleeding were defined as cases, patients with colonic diverticulosis were defined as controls, and the relationships with clinical findings were examined. To determine the risk factors for colonic diverticular bleeding, we estimated the odds ratio (OR) and 95% confidence interval (CI). Age was compared between groups using the Mann–Whitney *U* test, while frequency distributions were compared using the chi-square test or Fisher’s exact test. In multivariate analysis, we used a multiple logistic regression model with factors that had *p* values <0.2 on univariate analysis. A value of *p* < 0.05 was considered statistically significant. All statistical analyses were performed using Stata version 10 software (StataCorp, College Station, TX, USA).

## Results

During the study period, 3,722 patients underwent colonoscopy (Fig. 1). Of these, 903 patients participated in medical interviews (Fig. 1). Of the 903 patients, 645 patients had no colonic diverticula. Ultimately, 254 patients were included for analysis, comprising 45 patients with colonic diverticular bleeding and 209 patients with colonic diverticulosis. Table 1 shows the patient characteristics. Many of the patients were elderly men. Diverticulum location was predominantly right-sided or bilateral. Diverticulum type was cluster type in about half of the cases.

**Table 1:** Demographic of patient’s characteristics

	Patients
Median age (IQR)	67 (60–73)
Gender	
Male	178
Female	76
Location of diverticulum^a^	
Right side	125
Left side	59
Bilateral	70
Type of diverticulum^b^	
Sporadic/scatter type	135
Cluster type	119

^a^Right side, transverse and proximal colon; left side, descending and distal colon; bilateral, around the entire colon

^b^Sporadic type, 1; scatter type, 2–9; and cluster type, ≥10

Bilateral diverticula (OR, 4.5; 95% CI, 2.1–9.6), cluster type (OR, 5.3; 95% CI, 2.4–13), hypertension (OR, 3.3; 95% CI, 1.5–7.1), ischemic heart disease (OR, 4.1; 95% CI, 1.8–8.9), chronic renal failure (OR, 11; 95% CI, 2.1–67), antiplatelet drugs (OR, 2.3; 95% CI, 1.1–4.8), and NSAIDs (OR, 2.7; 95% CI, 1.0–6.8) were significant risk factors for diverticular bleeding (Table 2). On multivariate analysis, cluster type (OR, 4.0; 95% CI, 1.8–8.9; *p* < 0.01), hypertension (OR, 2.2; 95% CI, 1.0–4.6; *p* = 0.05), ischemic heart disease (OR, 2.4; 95% CI, 1.1–5.4; *p* = 0.03), and chronic renal failure (OR, 6.4; 95% CI, 1.3–32; *p* = 0.02) were identified as independent risk factors for diverticular bleeding (Table 3).

**Table 2:** Univariate analysis: risk factors for colonic diverticular bleeding

Factor	Cases (*n* = 45)	Controls (*n* = 209)	Odds ratio	95% CI	*p* value
Age					
<65	13	87	1 (referent)		
≥65	32	122	1.8	0.84–3.9	0.11
Gender					
Female	12	64	1 (referent)		
Male	33	145	1.2	0.57–2.8	0.60
Location of diverticulum^a^					
Left side	8	51	1 (referent)		
Right side	13	112	1.4	0.53–3.5	
Bilateral	24	46	4.5	2.1–9.6	<0.01
Type of diverticulum^b^					
Sporadic/scatter type	10	125	1 (referent)		
Cluster type	35	83	5.3	2.4–13	<0.01
Habits					
Alcohol drinking					
No	27	96	1 (referent)		
Yes	18	113	0.57	0.28–1.1	0.09
Smoking					
No	22	101	1 (referent)		
Yes	23	108	1.0	0.49–2.0	0.95
Comorbidities					
Hypertension					
No	13	119	1 (referent)		
Yes	32	90	3.3	1.5–7.1	<0.01
Hyperlipidemia					
No	34	175	1 (referent)		
Yes	11	33	1.7	0.71–3.9	0.17
Diabetes mellitus					
No	39	172	1 (referent)		
Yes	6	37	0.72	0.23–1.9	0.48
Ischemic heart disease					
No	28	182	1 (referent)		
Yes	17	27	4.1	1.8–8.9	<0.01
Chronic liver dysfunction					
No	43	195	1 (referent)		
Yes	2	14	0.65	0.069–3.0	0.75
Chronic renal failure					
No	39	206	1 (referent)		
Yes	6	3	11.0	2.1–67	<0.01
Medication					
Antiplatelet drugs^c^					
No	26	159	1 (referent)		
Yes	19	50	2.3	1.1–4.8	0.01
Aspirin	16	38			
Clopidogrel	2	1			
Cilostazol	1	1			
Ticlopidine	3	2			
Anticoagulants					
No	42	195	1 (referent)		
Yes	3	14	0.99	0.18–3.8	1.0
Acetaminophen					
No	45	205	1 (referent)		
Yes	0	4	0	0–4.5	1.0
NSAIDs					
No	36	191	1 (referent)		
Yes	9	18	2.7	1.0–6.8	0.02
Steroids					
No	44	201	1 (referent)		
Yes	1	8	0.57	0.013–4.5	1.0
Symptom					
Constipation					
No	32	164	1 (referent)		
Yes	13	45	1.5	0.66–3.2	0.29

*CI* confidence interval, *NSAIDs* nonsteroidal anti-inflammatory drugs

^a^Right side, transverse and proximal colon; left side, descending and distal colon; and bilateral, around the entire colon

^b^Sporadic type, 1; scatter type, 2–9; and cluster type, ≥10

^c^Antiplatelet drugs, including aspirin and other antiplatelet drugs

**Table 3:** Multivariate analysis: risk factors for colonic diverticular bleeding

	Odds ratio	95% CI	*p* value
Cluster type	4.0	1.8–8.9	<0.01
Hypertension	2.2	1.0–4.6	0.05
Ischemic heart disease	2.4	1.1–5.4	0.03
Chronic renal failure	6.4	1.3–32	0.02

## Discussion

The present detailed prospective survey identified cluster-type diverticula, hypertension, ischemic heart disease, and chronic renal failure as risk factors for colonic diverticular bleeding. Typical diverticular locations differ between the Asian and Western populations [6]. In our study, diverticula were predominantly right-sided or bilateral, unlike the predominantly left-sided distribution seen in the Western countries. Bilateral [13] and left-sided [14] predominance have not been reported as risk factors for diverticular bleeding, and location was not found to be a significant risk factor for diverticular bleeding with predominantly right-sided bleeding in this study. On the other hand, the number of diverticula has not previously been noted as a risk factor, but as the number of diverticula increased, the risk of bleeding increased significantly in our study. This represents a new finding. We speculate that diverticular bleeding occurs due to rupture of exposed blood vessels inside the diverticulum. As the number of diverticula increases, the number of potentially exposed blood vessels may also increase, resulting in a greater likelihood of bleeding.

Under conditions of hypertension, increased pressure within exposed blood vessels may elevate the risk for bleeding [12]. Vascular endothelial injury and atheroma formation occur, leading to arteriosclerosis. This arteriosclerosis causes fragility of exposed blood vessels in the diverticula, which then may lead to bleeding.

In our study, ischemic heart disease was newly identified as a risk factor for diverticular bleeding. Ischemic heart disease is a condition that reflects arteriosclerosis [24].

A case report found that patients with chronic renal failure may develop diverticular bleeding [17]. Our case–control study is the first to identify chronic renal failure as a risk factor for diverticular bleeding. In chronic renal failure, blood vessels throughout the body, including the intestine, are affected by arteriosclerosis [18–20]. In addition, heparin, which is used in dialysis, and platelet function also influence bleeding. Renal failure in association with arteriosclerosis and a bleeding tendency would thus represent a risk factor for diverticular bleeding. Patients with hemodialysis and chronic renal failure are at an increased risk for bleeding, and in such patients, caution should be exercised.

As with hypertension, ischemic heart disease, and chronic renal failure, rupture of exposed blood vessels in diverticula may occur due to arteriosclerosis. Use of antiplatelet drugs has often been reported as a risk factor for lower GI bleeding [21]. However, in reports on diverticulosis and diverticular bleeding, as in the present study, antiplatelet drugs have not been identified as an independent risk factor on multivariate analysis [12, 13]. Antiplatelet drugs, including aspirin, often are reported to cause mucosal injury of the upper GI tract and the small intestine [22, 23], but to the best of our knowledge, mucosal injury, such as ulcer formation in the colon, has not been reported. These drugs may thus also have few effects on blood vessels within diverticula. The influence of antiplatelet drugs on colonic diverticulosis should further be investigated in studies with a suitable sample size and study design.

By inhibiting PG synthesis through blocking of cyclooxygenase-1 activity, NSAIDs decrease mucosal protective function. NSAID injury of the lower GI tract mucosa has often been reported [21], and NSAIDs are also thought to be a risk factor in colonic diverticular bleeding [12, 14]. However, NSAIDs were not a risk factor for bleeding in our study. This may be because we excluded patients with decreased daily activities, due to low back pain or arthritic pain, and patients who were unable to undergo colonoscopy. Analysis therefore could not be performed in patients taking large doses of NSAIDs over a long period for chronic low back pain, which may have led to some bias. To investigate whether NSAIDs represent a risk factor for diverticular bleeding, a study that includes patients with decreased activities of daily living should be conducted.

No studies on the relationship between constipation and diverticular bleeding have previously been reported. As constipation is a condition that causes increased intestinal pressure [6], we hypothesized it as a potential risk factor. In our study, constipation was not identified as a risk factor for bleeding. This also represents a new finding. Hard stools alone, with constipation, may not have influence on mucosal injury within diverticula.

Although endoscopic therapy, angiography, and surgery were performed for colonic diverticular bleeding, it was not an established standard therapy to prevent bleeding [7]. Recently, a case report of barium enema therapy [26] and a case series of endoscopic band ligation therapy [27] were reported to effectively prevent colonic diverticular bleeding. Hypertension and chronic renal failure, which we determined as risk factors in this study, were also risk factors for rebleeding [10]. Therefore, our findings helped in the selection of high-risk patients who required these novel therapies in order to prevent bleeding. However, a limited number of studies on therapies for rebleeding have been reported. The effect of these therapies on colonic diverticular bleeding should be further investigated.

The limitation of this study was that patients in poor general health, for whom obtaining a medical history or performing colonoscopy was difficult, were not included. This may have introduced selection bias. To generalize the results of our study, a future multicenter cohort study would be desirable.

Hypertension, ischemic heart disease, and chronic renal failure are common diseases in daily clinical practice. In patients with colonic diverticulosis, the presence of these comorbidities must be considered in the risk for bleeding. Colonic diverticulosis is primarily a disease of the elderly and is a common disease encountered not only by gastroenterologists but also by physicians in general clinical practice (including cardiologists and nephrologists). Our results thus provide useful information for general medical care settings. Cluster-type diverticula, hypertension, ischemic heart disease, and chronic renal failure represent independent risk factors for colonic diverticular bleeding. This study identified new risk factors for colonic diverticular bleeding.

## Abbreviations

CI: Confidence interval
GI: Gastrointestinal bleeding
NCGM: National Center for Global Health and Medicine
NSAIDs: Nonsteroidal anti-inflammatory drugs
OR: Odds ratio
PG: Prostaglandin
UMIN: University Hospital Medical Information Network
